# *Stenotrophomonas maltophilia *in the respiratory tract of medical ICU patients

**DOI:** 10.1186/cc9602

**Published:** 2011-03-11

**Authors:** B Saugel, K Eschermann, R Schmid, W Huber

**Affiliations:** 1 Klinikum Rechts der Isar, München, Germany

## Introduction

*Stenotrophomonas maltophilia *can cause pneumonia in critically ill patients. The aim of the study was to investigate characteristics of critically ill patients with *S. maltophilia *isolated from the respiratory tract and to identify risk factors for *S. maltophilia *pneumonia and ICU mortality and to analyze antibiotic susceptibility of *S. maltophilia*.

## Methods

A retrospective analysis of medical records (November 2005 to December 2009) for three medical ICUs in a university hospital.

## Results

Sixty-four patients with *S. maltophilia *isolated from the respiratory tract (median age 66.0 years). Thirty-six patients fulfilled the criteria for diagnosis of pneumonia. Mechanical ventilation was needed in 51 patients. A significantly higher lung injury score was observed in patients with pneumonia compared with patients with colonization (*P *= 0.010). Independent risk factors for *S. maltophilia*-related pneumonia were higher Sequential Organ Failure Assessment (SOFA) score (*P *= 0.009) and immunosuppression (*P *= 0.014). Patients with *S. maltophilia *pneumonia had higher ICU mortality within a follow-up of 28 days (*P *= 0.040) and higher hospital mortality (*P *= 0.018) than patients with colonization. The highest antibiotic susceptibility rates were observed to trimethoprim-sulfamethoxazole, tigecycline, and moxifloxacin. A higher SOFA score when *S. maltophilia *was isolated (*P *= 0.001) and development of renal failure (*P *= 0.021) were independent risk factors for ICU mortality.

## Conclusions

Higher SOFA score and immunosuppression are independent risk factors for *S. maltophilia *pneumonia. Patients with pneumonia caused by *S. maltophilia *have a significantly higher ICU mortality within a follow-up of 28 days, hospital mortality and lung injury score compared with patients with *S. maltophilia *colonization.

